# Prediction of Enzyme Mutant Activity Using Computational Mutagenesis and Incremental Transduction

**DOI:** 10.1155/2011/958129

**Published:** 2011-10-09

**Authors:** Nada Basit, Harry Wechsler

**Affiliations:** Department of Computer Science, George Mason University, 4400 University Drive, Fairfax, VA 22030, USA

## Abstract

Wet laboratory mutagenesis to determine enzyme activity changes is expensive and time consuming. This paper expands on standard one-shot learning by proposing an incremental transductive method (T2bRF) for the prediction of enzyme mutant activity during mutagenesis using Delaunay tessellation and 4-body statistical potentials for representation. Incremental learning is in tune with both eScience and actual experimentation, as it accounts for cumulative annotation effects of enzyme mutant activity over time. The experimental results reported, using cross-validation, show that overall the incremental transductive method proposed, using random forest as base classifier, yields better results compared to one-shot learning methods. T2bRF is shown to yield 90% on T4 and LAC (and 86% on HIV-1). This is significantly better than state-of-the-art competing methods, whose performance yield is at 80% or less using the same datasets.

## 1. Introduction

A chain of amino acids in a given sequence forms the primary structure that makes up a protein and determines its functions. Proteins are necessary for virtually every activity in the human body [1]. There are twenty distinct amino acids that make up the polypeptides. They are known as proteinogenic or standard amino acids [1, 2]. The order of these amino acids in the chain, known as the primary sequence, is very important. Changes in even one amino acid (e.g., substituting one kind of amino acid, at a given location, with a different one) can affect the way the protein functions, that is, its activity. Such a substitution is an example of a mutation in the protein's amino acid sequence and is characteristic of a single-site mutation. 

The interplay between mutations and their effect on protein function is the domain of bioinformatics, in general, and computational mutagenesis, in particular. Mutagenesis can be described as creating a mutation in the protein (in the amino acid chain) by substituting an original (or wild-type) amino acid at a given position in the chain with one of the other 19 amino acid types, for example, substituting the amino acid tryptophan at position 10 with cysteine at that same location in a particular protein [3]. The resulting mutated protein's activity may be different from its wild-type counterpart (remaining active or becoming inactive). Experiments using mutagenesis enable researchers to collect data about protein activity with respect to mutations. Since wet lab experimentation is very expensive, finding a less expensive method, by being able to predict a protein's activity/function, is essential for both learning the range and scope of computational mutagenesis and drug design [4]. Automating this prediction task, that is, being able to perform protein function prediction in silico with the help of computational methods, is referred to as computational mutagenesis and is the topic for this article. The challenges faced in protein function prediction during in silico mutagenesis experiments and their validation include (i) annotation of large amounts of unlabeled biological data; and (ii) dealing with lack of consensus regarding proper labeling (“classification”) and consequent error propagation during data streaming and/or distributed annotation. The last challenge stands in contrast to classical one-shot classification and k-fold cross-validation where all the data, both labeled and unlabeled, become available and used at once for training, tuning, and testing. This paper builds on the protein representation proposed by Masso and Vaisman [5, 6]. Towards that end we propose to couple the expressive power of computational geometry and 4-body statistical potential for protein representation, with the robustness of statistical learning. In particular we use transduction, as the learning method of choice for protein function prediction, with enzyme mutant activity as the functionality of interest here. The datasets used come from the Protein Data Bank (PDB) [7], and the specific protein datasets used are HIV-1 protease, T4 Lysozyme, and Lac Repressor.

The outline of the paper is as follows Section 2 briefly surveys proteins, protein structure, and the relevance of protein mutations (Section 2.1). It also covers representational aspects including feature extraction, which are driven by computational geometry and 4-body statistical potential, and computational mutagenesis (Section 2.2).  Section 3 is about transduction while Section 4 describes a number of prediction methods and training strategies to be used for comparative evaluation. Experimental design, discussed in Section 5, includes descriptions of the datasets, protocols, and software used. Experimental results including comparative performance evaluation are presented and discussed in Section 6. The paper concludes in Section 7 with a summary of the contributions made and venues for future research.

## 2. Background

The relevance of mutagenesis is straightforward. As an example, let us consider sickle-cell anemia. It is an autosomal recessive genetic blood disorder affecting red blood cells, which is caused by a single error in the gene for hemoglobin. The incorrect amino acid at one position in the molecule causes the normally lozenge-shaped red blood cells to become rigid, and take the form of a sickle. This leads to a number of complications and shortens life expectancy to 42 in males and 48 in females [8]. We note for completeness that a mutation, by definition, is not limited to a change in only a single amino acid. Multiple site mutations, also known as multiple-point mutations, can occur when more than one amino acid mutates. This paper considers only single-site mutations.

### 2.1. Proteomics and Mutagenesis

Each residue (amino acid) is considered as a single point in the representation. Within a protein there are on the order of 10^3^ residues and there are on the order of 10^1^ residue chains. The largest hierarchical structure is the protein itself. Amino acids are critical to life and have many functions in metabolism. One of their most important functions is to serve as the building blocks of proteins, which are linear, unbranched, chains of amino acids. These molecules contain the basic elements of carbon, hydrogen, oxygen, and nitrogen [9, 10]. There are twenty distinct amino acids naturally incorporated into proteins [11]. As building blocks, proteins are chemically defined by the order of amino acid residues, and their primary structure. This in turn, determines their secondary, tertiary, and quaternary structure. Changes in amino acid sequence or structure may or may not affect the function of the protein.

### 2.2. Protein Representation Using Computational Geometry and 4-Body Statistical Potential

Given a protein and its amino acid sequence, one can represent it using methods drawn from computational geometry. Towards that end one considers each residue as a single point in 3D space using numerical coordinates, with the whole protein then represented by a 3D graph where the nodes are the amino acids and the edges connect to the nearest amino acids. The basic stages for protein representation include (i) protein space derivation (see Section 2.2.1), (ii) wild-type enrollment (see Section 2.3), and (iii) mutation representation (see Section 2.3.1). Once a protein is represented numerically/graphically one extracts features, which later on serve for classification of unlabeled mutants. Note that mutations are the result of editing the original amino acid sequence using substitutions; mutations are guided if the goal is drug design and synthesis. 

#### 2.2.1. Protein Space Derivation

The derivation of suitable protein representations for the purpose of function prediction starts with drawing a large sample of proteins from the Protein Data Bank (PDB) [7]. Amino acids are 3D structures and for the purpose of further processing they are abstracted in terms of their alpha carbon atomic coordinates. Each protein, that is, an amino acid sequence, is thus a sequence of corresponding alpha carbons (“C-alpha trace” or “backbone”). The sequence is subject to Delaunay tessellation, which yields Delaunay simplices in 3D and establishes nearest-neighborhood relationships between the amino acids making up the protein. One gathers “observed” statistics on all the Delaunay simplices from each amino acid sequence drawn from the PDB and compares them to the “expected” statistics gathered from all the Delaunay simplices corresponding to all possible combinations of amino acid vertices [5]. The ratio of the “observed” to “expected” statistics yields a log-likelihood score that is referred to as the 4-body statistical potential. This is described next.

**Delaunay Tessellation**

Over 1400 examples of different high-resolution crystallographic protein structures [5] with low primary sequence identity (different enough such that regions of the primary sequence do not match) are chosen from the PDB [5, 7]. The examples are used to obtain statistics needed for the 4-body statistical potential (see the following). Using the PDB coordinates for the amino acids, each protein is represented as a discrete set of points in 3D, corresponding to alpha carbon (C-alpha trace) atomic coordinates of each of the constituent amino acid [12, 13]. Choosing 3D coordinates based upon a weighted center of mass is another way to establish a single point to represent the amino acid in 3D. It is to be noted that a conscious decision has been made to mimic the same protein representation (that is using the alpha carbon) in our methods (see Section 4). The alpha carbon representation (C-alpha trace) is a good way to represent the protein structure, due to increased stability with respect to amino acid type. Another reason for using this representation is to provide identical conditions for a fair comparison between the method described by Masso et al. [12, 13] and the methods presented in this paper.

**Figure 1:**
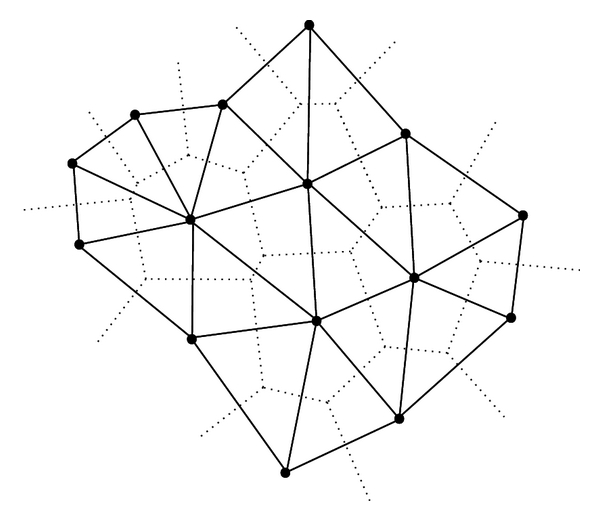
Delaunay tessellation (solid) and Voronoi Diagram (dotted) in 2D [16].

Delaunay tessellation of each protein structure yields an aggregate of nonoverlapping, space-filling, irregular tetrahedra (referred to as Delaunay simplices) whose vertices are the amino acid point representations [5, 12, 14]. The Quickhull algorithm performs the Delaunay tessellations [15]. A suite of Java and Perl programs is used to preprocess the PDB structure files, which includes checking for the absence of gaps (missing coordinates), and postprocessing (tabulation and calculating summary statistics) of the Quickhull output data (see Figure 1 for 2D Delaunay tessellation and its dual, the Voronoi diagram).

Each Delaunay simplex in a protein structure Delaunay tessellation objectively defines the nearest-neighbor amino acids for each given amino acid. The Delaunay simplex consists of four amino acids, which define its vertices. A significant property of this tessellation method is that the number of nearest neighbors in 3D is always four, which represents a fundamental topological property of 3D space. This is why Delaunay simplices are also known as quadruplets. While a given point (amino acid) can be a member of many quadruplets, all amino acid members in a given (one) quadruplet are called “nearest neighbors.” Assuming order independence within each quadruplet, the theoretical maximum number of all possible combinations of quadruplets that can be formed from the 20 amino acids naturally occurring in proteins is 8855 (disregarding the length of the sequence) [5, 12, 14].

**The 4-Body Statistical Potential**

After individually tessellating each of the sampled protein structures from the PDB, the observed frequency (*f*_*ijkl*_) of quadruples representing each quadruplet type across tessellations is calculated. A rate expected by chance (*p*_*ijkl*_; “expected frequency”) for each quadruplet based on a multinomial reference distribution is also calculated. Using the inverse Boltzmann law, the 4-body statistical potential function is an “empirical potential of quadruplet interaction (log-likelihood score) calculated as the logarithm of the ratio of observed normalized frequency to the expected chance of occurrence for every quadruplet” [5, 6, 12, 14]. Specifically, the log-likelihood score for a quadruplet (*i, j, k, l*) of amino acids is *q*_*ijkl*_ = log  (*f*_*ijkl*_/*p*_*ijkl*_). Here, *f*_*ijkl*_ is the observed normalized frequency of occurrence of quadruplets with vertices representing amino acids *i, j, k, l* among all the quadruplets formed by the tessellations of the training set of proteins, and *p*_*ijkl*_ = *ca*_*i*_ *a*_*j*_ *a*_*k*_ *a*_*l*_ is the expected rate of occurrence of the same quadruplet calculated from the multinomial distribution [5]. In the formula for *p*_*ijkl*_, *a*_*r*_ (where *r* = {*i*, *j*, *k*, *l*}) represents the normalized frequency of occurrence of the amino acid *r* among all of the training set proteins. If there are fewer than four distinct types of amino acids in the quadruplet, the formula will contain fewer than four *a_r_* factors. Similarly, the number of factors in the denominator of the permutation factor *c* = 4!/∏(*t*_r_!) depends on the number of distinct residue types that form the quadruplet, where *t*_r_ represents the number of residues in the quadruplet that are of type *r* [5]. Note that the statistical analysis of the residue composition of Delaunay induced quadruplets exhibits nonrandom preferences for certain quadruplets of amino acids to be clustered together [14]. This nonrandom preference motivates the benefit of developing the 4-body statistical potential that can be used in evaluating sequence-structure compatibility for the purpose of functional prediction [14]. The Auto-Mute website [5], run by Masso and Vaisman, provides further information on the data.

### 2.3. Wild-Type Enrollment

A protein structure that does not have any mutations is known as wild type (or wt). The 4-body statistical potentials are used to assign to individual amino acids a numerical value called the residue environment score. It is calculated by summing the log-likelihood scores of only those quadruplets in the Delaunay tessellation for which the point representing the amino acid position participates as a vertex [5, 17]. Collectively, the vector of residue environment scores for all of the amino acids in a protein is referred to as the potential profile of the protein.

#### 2.3.1. Mutation Representation

**Figure 2:**
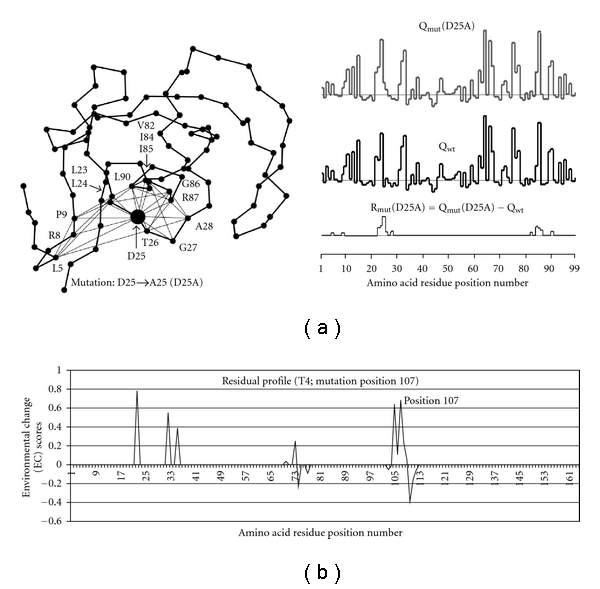
(a) An example of a C-alpha trace of a protein with the mutated position indicated (left); the potential profile for the mutated (right, top) and wild-type protein (right, middle) and the residual profile (right, bottom, figure adapted from Masso [18]). (b) Graphical representation of an input vector (residual profile) resulting from the mutation of the T4 lysozyme protein at position 107. Note the sparse nature of the vector; there are lots of positions with zero values and very few non-zero values.

Similar to wild-type enrollment one now derives in a similar fashion the potential profile for each mutation. A mutation is defined [5] by “utilizing the tessellation of the wild-type structure while substituting only the amino acid label at the CM (center of mass) point (vertex) representing the amino acid position of interest.” This only alters the residue environment scores of the mutated residue position as well as those residue positions whose respective points participate as vertices in quadruplets with the point representing the mutated residue position [5, 17] (The only log-likelihood scores that changed are of those quadruplets that have the mutated point as a vertex; all other scores remain unchanged). The residual profile vector of a protein mutant is defined as the difference between the mutant and wt protein potential profile vectors, and the value of each component is referred to as an environmental change (EC) score (see Figure 2). Hence, components with nonzero EC scores in the residual profile of a mutant identify the mutated position and all of its structural nearest neighbors.

## 3. Transduction

Transduction is different from inductive inference. It is local inference (“estimation”) that moves from particular(s) to particular(s) [19, 20]. In contrast to inductive inference, where one uses empirical data to approximate a functional dependency (the inductive step (that moves from particular to general)) and then uses the dependency learned to evaluate the values of the function at points of interest (the deductive step (that moves from general to particular)), one now directly infers (using transduction) the values of the function only at the points of interest from the training data [21, 22]. Inference takes place using both labeled and unlabeled data, which are complementary to each other. Transduction incorporates unlabeled data, characteristic of test (“query”) samples, in the classification process responsible to label them for the purpose of prediction. It further seeks for a consistent and stable labeling across both (near-by) training (“labeled”) and test data. Transduction seeks here to authenticate mutations whose function, for example, activity, is unknown, in a fashion that is most consistent with the given activities of known but similar protein and/or their mutations from the PDB. The search for putative labels (for unlabeled samples) seeks to make the labels for both training and test data compatible or equivalently to make the training and test error consistent.

Transduction “works because the test set provides a nontrivial factorization of the (discrimination) function class” [22]. One key concept behind transduction (and consistency) is the symmetrization lemma [23], which replaces the true (inference) risk by an estimate computed on an independent set of data, for example, unlabeled or test data, referred to as “virtual” or “ghost samples.” The simplest realization for transductive inference is the method of k-nearest neighbors. The Cover-Hart theorem [24] proves that asymptotically, the one nearest neighbor classification algorithm is bounded above by twice the Bayes' minimum probability of error. Similar and complementary to transduction is semisupervised learning (SSL) [23]. El-Yaniv and Gerzon [25] make useful analogies between “transduction and a “take-home exam” (where the student gets to see the questions and prepare accordingly), and between semi-supervised learning and a standard “classroom exam” where the student gets to see only exam questions from previous years before studying for the exam.”

## 4. Learning Methods

Much of the research on learning, in general, and modeling and prediction for the purpose of protein function prediction, in particular, has been done using one-shot learning where all the data is available at once for both training (for the purpose of one-shot learning) and cross-validation (for the purpose of performance evaluation) using randomized partitions [5, 16, 26]. This section provides details first on the best learning methods used for training and validation for one-shot learning, and then introduces alternative methods for incremental learning. All but one of the methods considered (decision trees [27, 28]) are characteristic of voting or ensemble methods. The random forest classifier [28], characteristic of voting methods, consists of a collection of decision trees. It combines the predictions made by multiple decision trees (e.g., taking the mode of the results of the individual trees) to obtain the final label (“class”). 

AdaBoost (adaptive boosting) works by adaptively and iteratively resampling data to focus learning on those samples that the previous weak classifier (learner) has failed on and/or encountered hard labels [29]. AdaBoost will iteratively choose *T* effective features to serve as weak (“better than chance”) classifiers, whose group behavior is characteristic of a strong (“robust”) classifier. The mixture of experts or the final strong classifier H for our binary cases of interest (“active” versus “inactive” function) is

(1) $$H\left(x\right)=\mathrm{sign}\;\;\left(\overset{T}{\underset{t=1}{\sum }}\alpha_{t}h_{t(x)}\right),$$

where *α*_*t*_ are the weights (“confidence”) for each classifier and *h*_*t*_ are the weak classifiers.

LogitBoost, similar to AdaBoost, fits an additive logistic regression model to the training data (a regression algorithm to train the weak learner) [30]. An additive model is an approximation to a function *F*(*x*) of the form

(2) $$F\left(x\right)=\overset{M}{\underset{m=1}{\sum }}c_{m}f_{m}\left(x\right),$$

where *M* is the number of boosting iterations, *c*_*m*_ are the weights, and *f*_*m*_ corresponds to weak classifiers. The algorithm minimizes the logistic loss:

(3) $$\sum \log\;\;\left(1+e^{-yF(x)}\right),$$

with *y* the true class label.

Another method used for comparison is support vector machines (SVM). It is characteristic of statistical learning [22, 31] and is known to be similar in scope and functionality to AdaBoost.

### 4.1. One-Shot and Incremental Transduction

We advance and describe here an alternative approach for protein function prediction, in general, and enzyme mutant activity, in particular. Towards that end, we take advantage of the statistical learning framework [22], and use transduction for prediction purposes. Several transductive strategies, both one shot (T1 [32]) and incremental (T2a, and T2b), are described below:

Data = L ∪ Q,
“L” = training (known labeled “function” mutants) set,
“Q” = test (unknown “function” mutants) set,
Inductive binary (−1, + 1) classifiers (C) = {Multi – Layer Network (MLN) trained using Back Propagation (BP) or Random Forest (RF)}.

#### 4.1.1. Strategy T1 [32]—One Shot Transduction

Data = L ∪ Q.

**Loop:**

- Train C on L.
- Make predictions on all Q using C such that for each unlabeled q (of Q) label (q) = h (C (q)) using the tan-sigmoid function h whose range is (−1, 1).
- L = L ∪ labeled  (Q).
- Iterate until convergence or some other “stopping criteria”.

Stopping criteria, for example, maximum number of iterations reached, or no changes in predicted labels have been made, from one iteration to the next one. 

#### 4.1.2. Strategy T2a—Incremental Transduction Using Annotation with Shrinking Test Set

Data = L ∪ Q

**Loop:**

- Train C on L.
- Make predictions on all Q using C such that for each unlabeled q (of Q) label (q) = h (C (q)) using the tan-sigmoid function h whose range is (−1, 1).
- Extract reliable labeled examples from Q as Q1. Q1 are examples whose continuous labels are greater in absolute value than 0.8.
- L = L ∪ Q1. The training set is augmented by Q1.
- Q = Q − Q1. The test set shrinks.
- Iterate until no “reliable” labels are found or test set (Q) becomes empty.

#### 4.1.3. Strategy T2b—Incremental Transduction Using Annotation with Size of Test Set Fixed

Data = L ∪ Q ∪ P,
Size of test set Q is empirically set to 80 examples,
“P” = test pool—a secondary and larger test set separate from “Q”.

**Loop:**

- Train C on L.
- Make predictions on all Q using C such that for each unlabeled q (of Q) label (q) = h (C (q)) using the tan-sigmoid function h whose range is (−1, 1).
- Extract reliable labeled examples from Q as Q1. Q1 are examples whose continuous labels are greater in absolute value than 0.8.
- L = L ∪ Q1. The training set is augmented by Q1.
- Q = Q − Q1. The test set shrinks.
- Q = Q ∪ P1. The test set is replenished by extracting some unlabeled examples from P as P1, such that |Q| = 80 stays constant.
- Iterate until no “reliable” labels are found or test set (Q) and/or test pool (P) become empty.

## 5. Experimental Design

The mutations under consideration for the purpose of enzyme mutant activity predictions are those of HIV-1 protease, bacteriophage T4 lysozyme, and Lac repressor. Data comes from the RCSB Protein Data Bank (PDB) (http://www.pdb.org), which is an international repository for processing and distribution of 3D macromolecular structure data, and is primarily determined experimentally. The Delaunay tessellations of HIV-1 protease, bacteriophage T4 lysozyme, and Lac repressor are based on the structural coordinates obtained from PDB accession files 3phv, 3lzm, and 1efa, respectively. The data is fed into the learning algorithms (see Section 4) in the form of residue profile vectors (see Section 2.3) courtesy of Masso and Vaisman [5, 26]. Prediction concerns activity, which is related to some particular function, and is characterized using binary labels. If a protein (wt or mutant) is carrying out some particular function at an acceptable level, compared to some predefined threshold, then the protein's activity is considered “active” (+1). If a protein, due to a mutation or otherwise, does not perform the same particular function at an acceptable level (with respect to the wt protein or otherwise) or ceases to function at all, then the protein activity is considered “inactive” (−1) [4]. Using the protein hemoglobin again as an example, its activity will be considered “active” if it is able to successfully transport oxygen from the lungs to the rest of the body (tissues) where it releases the oxygen for cell use, and collects carbon dioxide to return to the lungs. The hemoglobin's activity would be considered “inactive” if it was not able to perform this function or was not able to perform its function at the expected level of efficiency, for example, due to sickle-cell disease. For all the mutation examples in our datasets, the “ground truth” protein activity in each case has been experimentally determined in the lab and provides the binary class labels used for training and validation. The characteristics of the datasets are briefly described next.

**HIV-1 Protease**

It contains a single chain of 99 amino acid residues. There are 1881 possible single point mutations (mutants) that can be engineered (19  (alternative  mutations) × 99  (sites) = 1881). There is experimental activity data available for 536 mutants that are distributed among all 99 positions. For these 536 mutant HIV-1 protease enzymes, the label for each one has been experimentally determined to be either “active” (224 samples) or “inactive” (312 samples) [5, 17].

**Bacteriophage T4 Lysozyme**

It contains a single chain of 164 amino acid residues. There are 3116 possible single-point mutations (mutants) that can be engineered (19 × 164 = 3116). There is experimental activity data available for 2015 mutants that are distributed between positions 2–164, and the dataset has 12–13 mutations at each of the positions. For these 2015 mutants, bacteriophage T4 lysozyme enzymes, the label for each one has been experimentally determined to be either “active” (1724 samples) or “inactive” (291 samples) [5].

**Lac Repressor**

It was tessellated from chain “B”, which consists of 331 amino acid residues. There are 6289 possible single-point mutations (mutants) that can be engineered (19 × 331 = 6289). There is experimental data available for 4041 mutants that are distributed among positions 2 and 329. The dataset has 12–13 mutations at each of the positions. For these 4041 mutant Lac repressor enzymes, the label for each one has been experimentally determined to be either “active” (2267 samples) or “inactive” (1774 samples) [13].

The protocols used for performance evaluation are as follows. The experiments (both one-shot and incremental learning) are carried out using 4-fold cross-validation when using the HIV-1 dataset and 10-fold cross-validation when using the T4 and LAC datasets. 10-fold cross-validation was not performed on the HIV-1 dataset since the size of this dataset is too small. An important experimental design implementation is that during cross-validation the folds are not randomly generated. Instead, a method of “smart partitions” is employed for all the learning algorithms compared. Towards that end, data is organized such that samples representing a mutation at a given position in the primary structure of the protein are evenly distributed (as much as possible) between each of the folds. This ensures that during cross-validations there would always be samples of mutations at the same locations for both training and testing. 

Two kinds of experimental designs are carried out. The first set of experiments involves one-shot learning and classification, and explores the classification accuracies of five popular learning algorithms (AdaBoost, LogitBoost, SVM, random forest, and decision tree) as well as the standard transduction algorithm (Strategy T1). They are run in standard way with no additions or modifications. The second series of experiments involves the introduction of our novel incremental transductive strategies (Strategy T2a and T2b) where only “reliable” labeled samples augment the original training sets. The majority of the experiments and algorithms were written and ran using MATLAB [33], with the remaining ones using WEKA [34]. Microsoft Excel was used to aid in preprocessing the data. Other preprocessing and data manipulation programs were written to aid in other miscellaneous tasks (such as to format the data to suit the input style of the novel algorithms), and these were written and run using MATLAB.

## 6. Experimental Results

All the experiments were run using the three datasets described in the previous section, that is, HIV-1, T4, and LAC. 4-fold and 10-fold cross-validation is employed using smart (balanced) partitioning. The results reported are based upon an average of 10 runs using four performance evaluation indexes: average accuracy, standard deviation, sensitivity, and specificity. Confusion (“contingency”) matrices are derived for protein “binary” function (“activity”) prediction for each experiment using different learning algorithms. Using TP, TN, FP, and FN to indicate true positive, true negative, false positive, and false negative rates, respectively, the performance indexes are defined as follows. Accuracy is defined as (TP + TN)/(TP + TN + FP + FN). Sensitivity (the true positive rate), which is defined as TP/(TP + FN) is a measure of how well the positive class is predicted. A test with high sensitivity has a low Type II error rate. While a good and useful performance indicator, its sensitivity does not describe how well predictions are made for the other classes, in this case the negative class. Towards that end, specificity (the true negative rate) is defined as TN/(TN + FP). A test with high specificity has a low Type I error rate.

The first series of experiments explores the classification accuracy of five popular learning algorithms, AdaBoost, LogitBoost, SVM, RF (random forest), and DT (decision tree), as well as the standard transduction algorithm (Strategy T1 using neural networks or random forests as base classifier; see Section 4.1). One can see from Table 1 that one-shot random forest (RF) performs best. One can also see that random forest (RF) as base classifier improves the performance of one-shot transduction (T1) compared to neural networks. These experiments implement a one-shot training and cross-validation methodology and are performed with the no-selectivity option, where all the test data gets labeled and augments the training set. Table 1 reports the outcomes for “traditional” one-shot methods for which balanced and randomized partitions yield similar results, which are consonant with the results reported by Masso and Vaisman [5, 13]. We note that the random forest algorithm performs the best compared to the other classifiers including the standard transductive algorithm [32]. 

The next series of experiments employs incremental learning and validation methodology. As a consequence, there are errors in annotation, which possibly propagate during training. These experiments therefore assess how learning is impacted by adding possibly mislabeled examples to training data. Note that ground truth is always available but only for evaluation purposes. Towards that end the second series of experiments uses the transductive incremental strategies T2a and T2b (see Section 4.1) using selectivity, that is, only confidently labeled examples (but possibly incorrectly labeled) augment the training set.  Table 2 reports on incremental learning using balanced partitions and shows that the best results obtained using T2bRF are superior to those obtained under less stringent one-shot conditions (see Table 1).

The incremental transductive approach with a neural network (MLN) as the base classifier (Strategy T2aNN and T2bNN in Table 2) performs better than one-shot transductive T1 (see Table 1). The motivation for using random forests (RF) as a base classifier (Strategy T2aRF and T2bRF in Table 2) comes from Table 1 where it was shown to perform the best for all three datasets. The methodology behind the results in Table 2 (incremental transductive learning) is more realistic and true to real-life circumstances than one-shot learning. This methodology closely resembles the situation with biological data where there is an abundance of unlabeled data and limited amounts of labeled data, with the latter slowly augmented by experimental results from the lab. It is now apparent that incremental transduction learning using selectivity yields better results than one-shot learning (compare Table 2 against Table 1). In particular we note that random forest was found to be the optimal base classifier for incremental transduction (using random forest with strategy T2b; See Strategy T2bRF in Table 2); their hybrid enzyme mutant activity prediction method, T2bRF, yields 86.88% on HIV-1, 90.97% on T4, and 90.84% on LAC. This compares favorably against an accuracy of 79.28% on HIV-1, 87.12% on T4, and 80.80% on LAC using standard methods (see Table 1). The results obtained using T2bRF are also significantly better than state-of-the-art competing methods reported in bioinformatics [5, 13], whose performance is 80% or less using the same datasets from PDB. The results are even more impressive when one considers that incremental learning is more stringent than one-shot learning. Note also that the ratio of “active” class to “inactive” class is very skewed (most data belongs to active class) for T4 compared to HIV-1 and LAC, which is the reason for better performance on T4 compared to LAC (see Tables 1 and 2). Tables 1 and 2 also show that there is a tradeoff between sensitivity and specificity regarding HIV when using one-shot and incremental learning. Regarding T4 and LAC Tables 1 and 2 also show a significant improvement on specificity from using incremental rather than one-shot learning. 

## 7. Conclusion

This paper expands on standard one-shot learning for enzyme mutant activity prediction using incremental learning. The computational approach proposed is driven by existing methods for protein sequence representation using Delaunay tessellation and 4-body statistical potential. The novelty of the paper comes from the use of transduction strategies for incremental learning. The use of random forest has been empirically found to perform best as base classifier for both one-shot learning and incremental learning. The novel enzyme mutant activity prediction method—T2bRF—driven by incremental transduction using random forests as base classifier, has been found empirically and cross-validated to compare favorably against current state-of-the-art contending methods. 

Venues for future research include (a) access to multiple protein and site mutations and their synergy, (b) alternative representational methods where the effect of mutations is fully reflected in updated Delaunay tessellations with optimal feature selection available, and (c) investigating additional functionalities, for example, protein stability (instead of protein activity).

## Figures and Tables

**Table 1:** One-shot learning.

Dataset	Algorithm	Avg. Acc.	St. Dev.	Sensitivity	Specificity
HIV-1	AdaBoost	68.84	4.77	0.87	0.63
	LogitBoost	75.93	3.90	0.72	0.86
	SVM	68.65	3.50	0.66	0.75
	RF	79.28	1.96	0.88	0.76
	DT	77.57	1.21	0.73	0.81
	T1 NN	73.13	2.57	0.69	0.76
	T1 RF	74.64	3.24	0.65	0.74

T4	AdaBoost	85.10	0.14	0.98	0.11
	LogitBoost	85.65	0.31	0.97	0.20
	SVM	86.88	0.24	0.99	0.17
	RF	87.12	0.44	0.97	0.30
	DT	85.33	0.56	0.93	0.34
	T1 NN	75.46	6.99	0.80	0.46
	T1 RF	85.02	7.44	0.94	0.35

LAC	AdaBoost	60.53	0.31	0.99	0.12
	LogitBoost	71.88	0.58	0.91	0.48
	SVM	72.15	0.16	0.88	0.52
	RF	80.80	0.37	0.86	0.75
	DT	78.71	0.34	0.83	0.74
	T1 NN	65.23	3.58	0.76	0.39
	T1 RF	77.73	3.64	0.78	0.77

Results of the AdaBoost, LogitBoost, SVM, random forest, decision tree, and transduction T1 algorithms. Using one-shot learning, no selectivity, and 4-fold cross-validation for the HIV-1 dataset and 10-fold cross-validation for T4 and LAC datasets.

**Table 2:** Incremental Transductive Learning.

Dataset	Strategy	Avg. Acc.	St.Dev.	Sensitivity	Specificity
HIV-1	T2aNN	75.53	2.70	0.71	0.76
	T2bNN	78.05	2.50	0.75	0.81
	T2aRF	83.46	2.62	0.78	0.82
	T2bRF	86.88	2.55	0.76	0.83

T4	T2aNN	82.69	4.11	0.89	0.50
	T2bNN	82.64	5.14	0.90	0.56
	T2aRF	89.71	3.54	0.93	0.63
	T2bRF	90.97	3.48	0.94	0.67

LAC	T2aNN	76.17	2.88	0.78	0.75
	T2bNN	82.51	2.93	0.80	0.75
	T2aRF	86.54	2.71	0.86	0.80
	T2bRF	90.84	2.87	0.86	0.80

Results of transductive learning algorithms T2a and T2b on HIV-1, T4, and LAC using incremental transductive learning, and selectivity. (the number of folds used for cross-validation is 4 for the HIV-1 dataset and 10 for T4 and LAC.)
